# Anchoring spinel cobalt and zinc ferrites on zeolite for highly synergic photocatalytic reduction of chromium (VI)

**DOI:** 10.1038/s41598-024-83427-y

**Published:** 2024-12-30

**Authors:** Moin Mehrbakhsh, Moones Honarmand, Ahmad Aryafar

**Affiliations:** 1 Department of Mining Engineering, Faculty of Engineering, University of Birjand, Birjand, Iran; 2 Department of Chemical Engineering, Birjand University of Technology, Birjand, Iran

**Keywords:** Cr(VI) reduction, Photocatalysis, Spinel ferrites, CoFe_2_O_4_–Zeolite–ZnFe_2_O_4_, Chemistry, Nanoscience and technology

## Abstract

To tackle the challenges of increasing the efficiency of photocatalysts, a ternary magnetic heterojunction photocatalyst containing spinel cobalt and zinc ferrites, and zeolite (CZZ) was designed and fabricated. The physicochemical properties of the novel photocatalyst were verified using characterization techniques such as XRD, FT-IR, FE-SEM, EDS mapping, N_2_ adsorption-desorption, VSM, PL, and UV–Vis DRS. The CZZ photocatalyst exhibited a significant Cr (VI) reduction rate of 0.1535 min^−1^, which was 9.27, 5.37 and 3.58 times higher than those of single ZnFe_2_O_4_ nanoparticles (0.0166 min^−1^), CoFe_2_O_4_ nanoparticles (0.0286 min^−1^), and CoFe_2_O_4_–ZnFe_2_O_4_ (0.0428 min^−1^) respectively. CZZ showed an excellent reusability after three consecutive cycles of Cr(VI) reduction. The results from the experiments in different aqueous environments displayed that CZZ could be a promising photocatalyst to reduce Cr(VI) in the treatment of actual aqueous matrices. The present study not only provides a highly active catalytic system for the practical removal of Cr(VI) but also paves the way for the fabrication of high-performance heterojunction photocatalysts.

## Introduction

Hexavalent chromium (Cr(VI)) as one of the most poisonous heavy-metal is abundantly found in wastewater from different industries such as steel and rubber manufacturing, chromium mining, electroplating, pigments, petroleum refining, and leather tanning^1,2^. Cr(VI) can be hazardous to human health. Excessive exposure to Cr(VI) can cause cancer, kidney and liver disease, and respiratory problems^3,4^. The World Health Organization (WHO) strongly recommends that the concentration of Cr(VI) in drinking water should not exceed 50 ppb^5^. Therefore, removing Cr(VI) from aquatic environments is necessary to maintain the health of humans and other living organisms. Because of the importance of the matter, different methods have been developed to treat Cr(VI), including adsorption^6^, electrochemical^7^, microorganism^8^, membrane separation^9^, etc. Still, they suffer from low efficiency, secondary pollution, and high costs. The shortcomings of these methods are not compatible with the principles of green and sustainable water purification.

Advanced oxidation processes (AOPs) are practical methods for efficiently removing pollutant and are also valuable options for wastewater treatment^10–12^. The difference between this technique and other methods is that all toxic pollutants are converted into non-toxic forms without changing the catalyst. AOPs include: Fenton^13^, ozonation^14^, sonolysis^15^ and photocatalysis reactions^16–18^. Among numerous sustainability approaches, the solar–driven photocatalytic processes have attracted much attention for removing containments due to their environmental friendliness, excellent efficiency, cleanliness, and low cost and energy consumption^19^. The photocatalytic processes use light energy to generate active species capable of mineralizing or reducing the target pollutants. Regarding the removal of chromium contaminants, photocatalytic technology can convert Cr(VI) to Cr(III) using photo induced reduction^20–26^. This process is valuable because Cr(III) is not only less toxic than Cr(VI), but also considers as an essential trace element for the human body^24^.

Since the advent of photocatalytic processes, various modifications have been made to increase their efficiency. Photocatalysts containing heterojunctions have become more prominent than other photocatalytic modification methods due to their high efficiency and reliability. Inspired by the photosynthesis of plants process, an exciting strategy of using two semiconductors to fabricate direct Z-scheme heterojunction has been developed for effective pollutant removal. However, due to insufficient knowledge about the transfer path of the photogenerated charge carriers between the semiconductors, the Z-scheme photocatalysts were mainly constructed by trial and error. Type II heterojunctions often replaced Z-scheme heterojunctions and then, as expected, lost their redox ability. The existing defects have been eliminated in S-scheme heterojunctions, so that by intelligent selection of semiconductors, unusable electrons and holes can be quickly recombined in the presence of an internal electric field, and holes and electrons those with more favorable redox potential remain^27–30^. As a result of this process, the photocatalytic performance is significantly improved. An attractive strategy for making S-scheme heterojunctions is combining two semiconductors^31,32^. Depending on the structural and optical properties of semiconductors, their application in photocatalytic reactions can be limited or extensive. Before designing heterojunctions, it should be noted that using photocatalysts on a real scale is still a severe problem because of their complex and time-consuming separation from the reaction mixture. Therefore, using magnetic semiconductors as an efficient approach leads to easy and fast separation and recovery of the photocatalyst. Among the available magnetic semiconductors, non-carcinogenicity spinel ferrites such as cobalt ferrite (CoFe_2_O_4_), and zinc ferrite (ZnFe_2_O_4_) show significant catalytic capabilities in photocatalytic processes. Spinel ferrites as ferromagnetic metal oxides, in addition to their simple separation which promotes principles of green chemistry, exhibits an acceptable photocatalytic performance due to their narrow band^33^. Most importantly, because of the strong bond between metal and iron, these compounds have high chemical stability and can withstand harsh reactions. Also, the stability of spinel ferrites in strongly acidic or alkaline environments makes them a suitable option for practical industrial applications^34^. The unique features of metal ferrites have caused their use in photocatalytic reactions to increase in recent years^35^. The presence of spinel ferrites in heterojunction structures increases the efficiency of the photocatalytic reaction by improving charge separation. Also, due to the excellent magnetic properties of ferrites, catalyst recovery is carried out more easily and quickly.

Along with all the advantages of spinel ferrites, their agglomeration is a severe problem due to their high magnetic properties. Also, the magnetic structure of spinel ferrites loses its efficiency over time because of the accumulation property^36^. Therefore, to exploit the maximum potential of magnetic nanoparticles, the problem of their lack of effective dispersion must be solved in some way. It is well known that anchoring nanoparticles on support is an effective strategy to prevent the aggregation of magnetic nanoparticles. Due to the effectiveness of this method, various types of porous materials such as cellulose^37^, carbon nanotube^38^, graphitic carbon nitride^34^, silica^39^, graphene oxide^36^, diatomite^40^, biochar^41^, etc. have been performed as carriers to stabilize spinel ferrites.

**Fig. 1:**
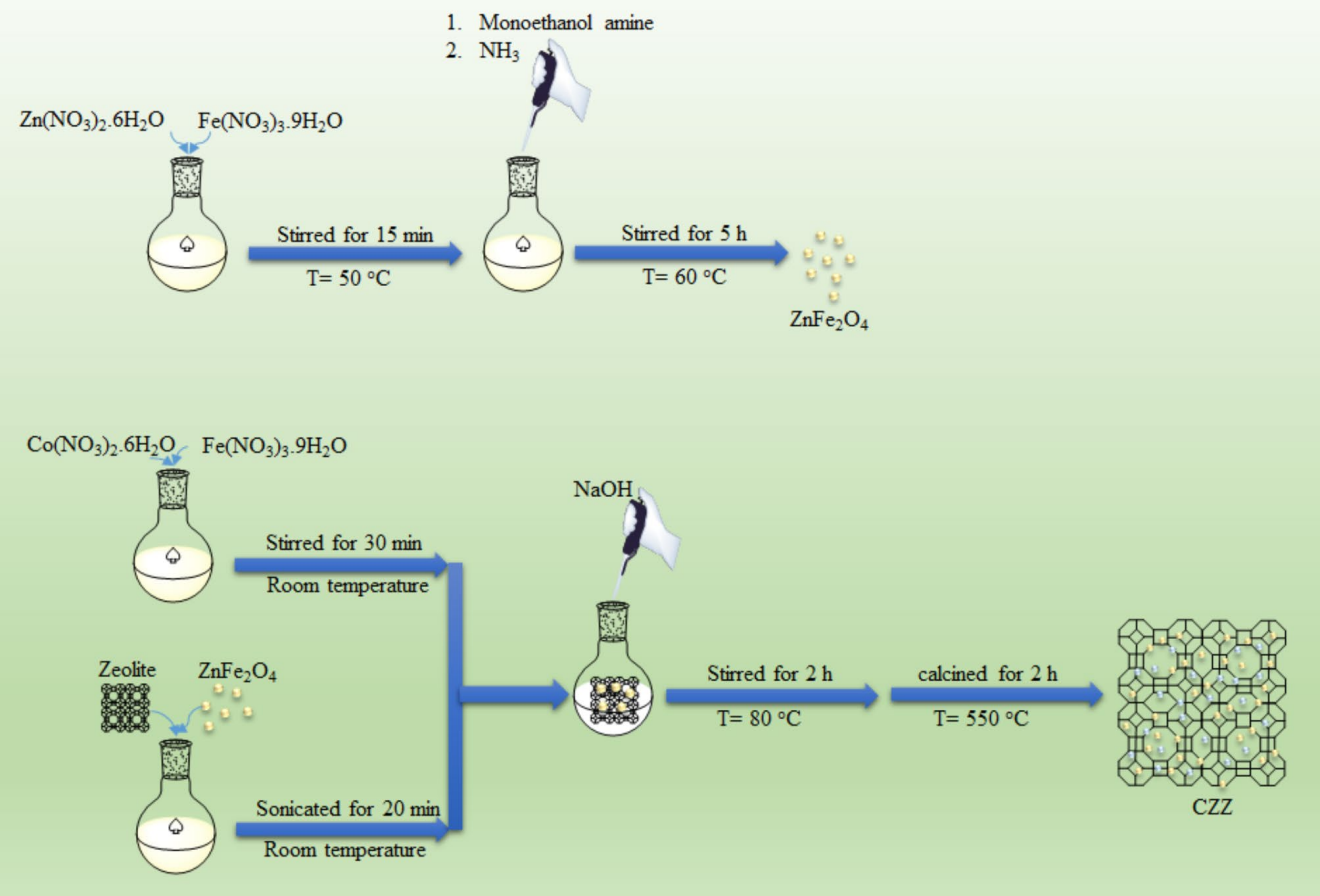
Schematic synthesis of CZZ photocatalyst.

In this study, inspired by the spinel ferrites, for the first time, CoFe_2_O_4_–Zeolite–ZnFe_2_O_4_ (CZZ) ternary photocatalyst was successfully designed and fabricated by a simple co-precipitation method (Fig. 1). After the characterization of CZZ using various techniques, its photocatalytic performance was evaluated for Cr(VI) reduction using renewable solar energy. To our knowledge, some papers have been published on reducing Cr(VI) using various photocatalysts. However, no published report so far about photoreduction of Cr(VI) using a ternary magnetic photocatalyst and natural sunlight. Expensive lamps with a limited lifespan have been used to stimulate the photocatalyst. One of the innovations of this study is that natural sunlight has been employed as a free and renewable source with unlimited lifetime and broad wavelength. Also, considering that the separation of photocatalysts is always a challenge, using magnetic materials is regarded as a suitable solution for photocatalyst recovery, which we achieved in this study. To achieve the best result, the influencing parameters on Cr(VI) reduction in CZZ system were investigated. Compared to single semiconductors, enhanced photocatalytic technology using heterojunction structures is an efficient purification technique.

## Experimental section

### Synthesis of catalysts

ZnFe_2_O_4_ was synthesized through the simple co-precipitation method. At first, aqueous solutions of Fe(NO_3_)_3_·9H_2_O and Zn(NO_3_)_2_·6H_2_O were prepared with 0.2 M and 0.1 M concentrations, respectively. Then, 25 mL of each solution was transferred to a round-bottomed flask and stirred vigorously at 50 °C for 15 min. Afterward, monoethanolamine (3 mL) was added drop wisely to the stirring solution. By adding a few drops of NH_3_, the pH of the solution was adjusted to about 10. Subsequently, the resulting solution was further stirred at 60 °C under refluxed conditions for 5 h. After cooling at ambient temperature, the obtained precipitates were separated from the reaction solution by an external magnet, washed twice with water and ethanol to remove impurities, and then dried at room temperature for 48 h.

For the fabrication of CZZ, 10 mL of each aqueous solution Fe(NO_3_)_3_·9H_2_O (0.2 M) and Co(NO_3_)_2_·6H_2_O with stoichiometric amounts 2:1, respectively, were mixed at laboratory temperature for 30 min (solution A). In another round-bottomed flask, distilled water (10 mL), zeolite (0.2 g), and ZnFe_2_O_4_ made in the previous step (0.4 g) were mixed and sonicated for 20 min (solution B). Then, the solution A and B were transferred to a giant round-bottom flask and subjected to vigorous stirring. NaOH solution was slowly added to the stirring solution to adjust its pH to above 12.0. The solution mixture was then treated for 2 h under reflux conditions at 80 °C. Similar to the previous step, the obtained precipitates were separated from the reaction solution by an external magnet, washed twice with water and ethanol to remove impurities and dried at room temperature for 48 h. To convert the precursors into the CoFe_2_O_4_–Zeolite–ZnFe_2_O_4_ nanocomposites, the formed precursors were calcined in an electrical furnace for 2 h at 550 °C. For comparative objectives, pure CoFe_2_O_4_ nanoparticles were synthesized similarly to CZZ without adding zeolite and ZnFe_2_O_4_.

### Photocatalytic tests

The photocatalytic performance of the synthesized photocatalysts was investigated through Cr(VI) reduction under actual solar irradiation conditions (Time: June to August 2023, 10:00 a.m. to 1 p.m.; weather: sunny; light intensity: ∼270 KLux). In a typical test, a certain amount of photocatalyst was added to 50 mL K_2_Cr_2_O_7_ solution. Aqueous solutions of HCl or NaOH were used to adjust the solutions pH. The mixture was stirred in darkness for 15 min to make saturated adsorption. The photocatalytic reaction was initiated by exposing the samples to direct sunlight. The concentration of Cr(VI) was monitored every 15 min with the 1,5-diphenylcarbazide method^42^ on a UV–Vis spectrometer and calculated by the below formula:

$$\:\text{D}\text{e}\text{g}\text{r}\text{a}\text{d}\text{a}\text{t}\text{i}\text{o}\text{n}\:\text{e}\text{f}\text{f}\text{i}\text{c}\text{e}\text{n}\text{c}\text{y}\:\text{\%}=\frac{{\text{C}}_{0}-\text{C}}{{\text{C}}_{0}}\:\times\:100$$

C_0_ and C are the initial concentration and concentration of Cr(VI) (ppm) at the reaction time t (min), respectively.

## Results and discussions

### Characterization of the samples

**Fig. 2:**
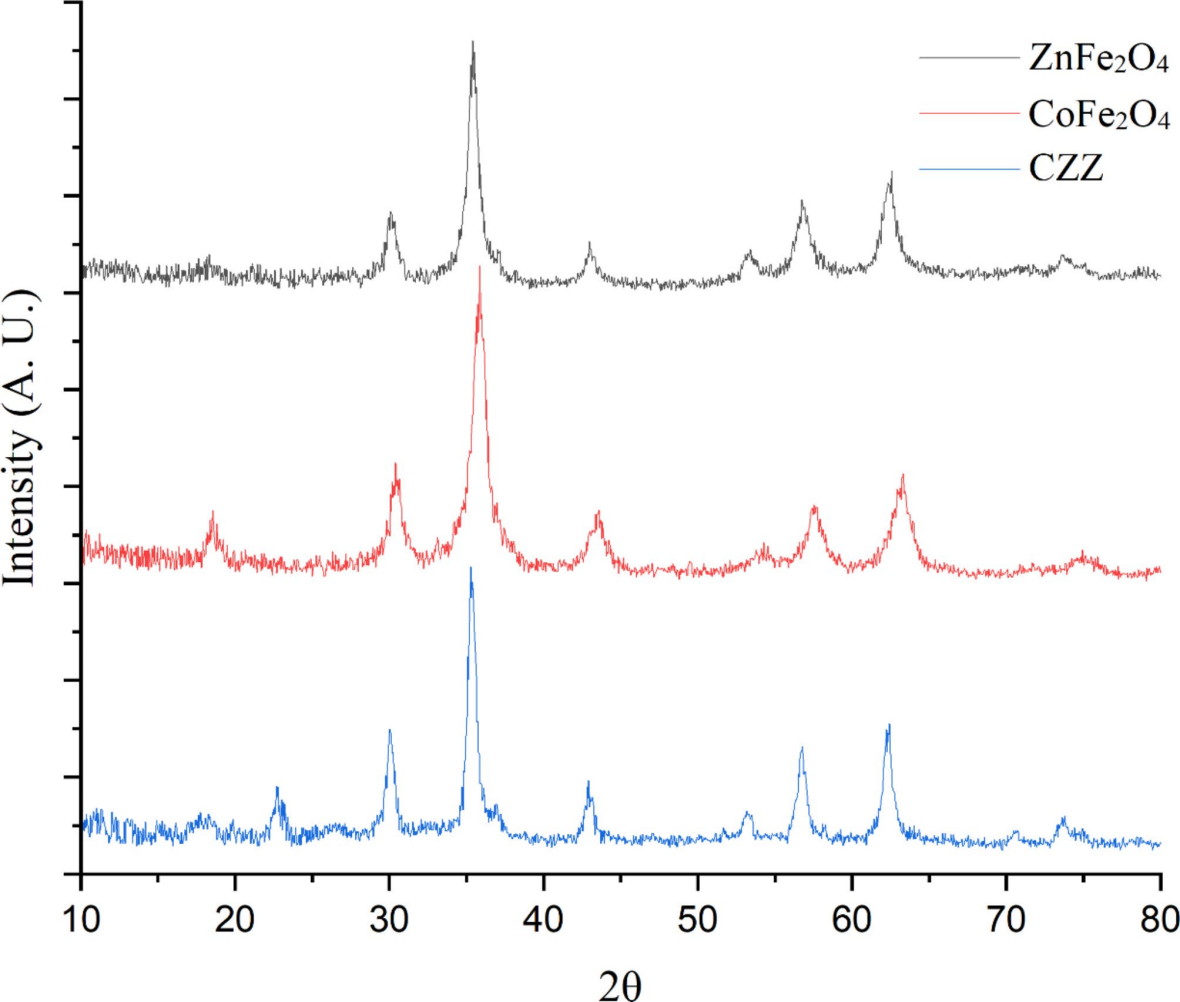
XRD patterns of spinel ZnFe_2_O_4_ and CoFe_2_O_4_ nanoparticles and CZZ.

XRD analysis is recorded to study the crystallinity and type of phase of the synthesized samples. The characterization of three materials viz., ZnFe_2_O_4_, CoFe_2_O_4_, and CZZ was carried out using XRD (Fig. 2). In the XRD pattern of pure ZnFe_2_O_4_ nanoparticles, the diffraction peaks at 2θ = 30.27°, 35.52°, 43.02°, 53.47°, 56.82°, 62.32° and 73.67° are ascribed to (220), (311), (400), (422), (511), (440) and (533) planes of spinel zinc ferrite (JCPDS. No. 89–1012)^43^, respectively. In addition, in the XRD pattern of CoFe_2_O_4_, eight diffraction peaks at 2θ = 18.62° (111), 30.52° (220), 35.87° (311), 43.82° (400), 54.52° (422), 57.37° (511), 63.42° (440) and 74.92° (533) matches well with the spinel cobalt ferrite (JCPDS no. 01-1121)^44^. The characteristic peaks of impurities do not reveal in XRD patterns of synthesized samples, which confirm the high purity of the structure of the ZnFe_2_O_4_ and CoFe_2_O_4_ nanoparticles. From further examination of the XRD patterns of the CZZ, it can be understood that the coupling of two semiconductors has caused to X-ray diffraction patterns move slightly to the lower angle, which could be related to partial substitution of cobalt atom (ionic radius Co^2+^, 0.072 nm) by the larger size of zinc atom (ionic radius Zn^2+^, 0.074 nm) at surface interaction. Also, as can be seen, the peak corresponding to 2θ = 18.62° of CoFe_2_O_4_ in the CZZ diffraction pattern is relatively broad, which is due to the overlapping the prominent peaks of zeolite with it. The Scherrer’s equation is utilized to calculate the size of the synthesized samples. Based on this equation, the dimension of nanoparticles is estimated to be 8 and 6 nm for ZnFe_2_O_4_ and CoFe_2_O_4_, respectively. Furthermore, the CZZ heterojunction demonstrates the typical patterns of ZnFe_2_O_4_ and CoFe_2_O_4_ without any apparent changes in their crystal structure, supports the successful fabrication of the nanocatalysts and the loading of spinal zinc and cobalt ferrites on zeolite surface with a reasonable degree of crystallinity. Also, in the XRD pattern of CZZ, the (101) diffraction plane at 2θ = 22.67° is the only characteristic peak attributed to zeolite. The non-appearance of other zeolite peaks is due to the low amount of used zeolite during nanocatalyst synthesis and the deposition of nanoparticles over zeolite sheets.

**Fig. 3:**
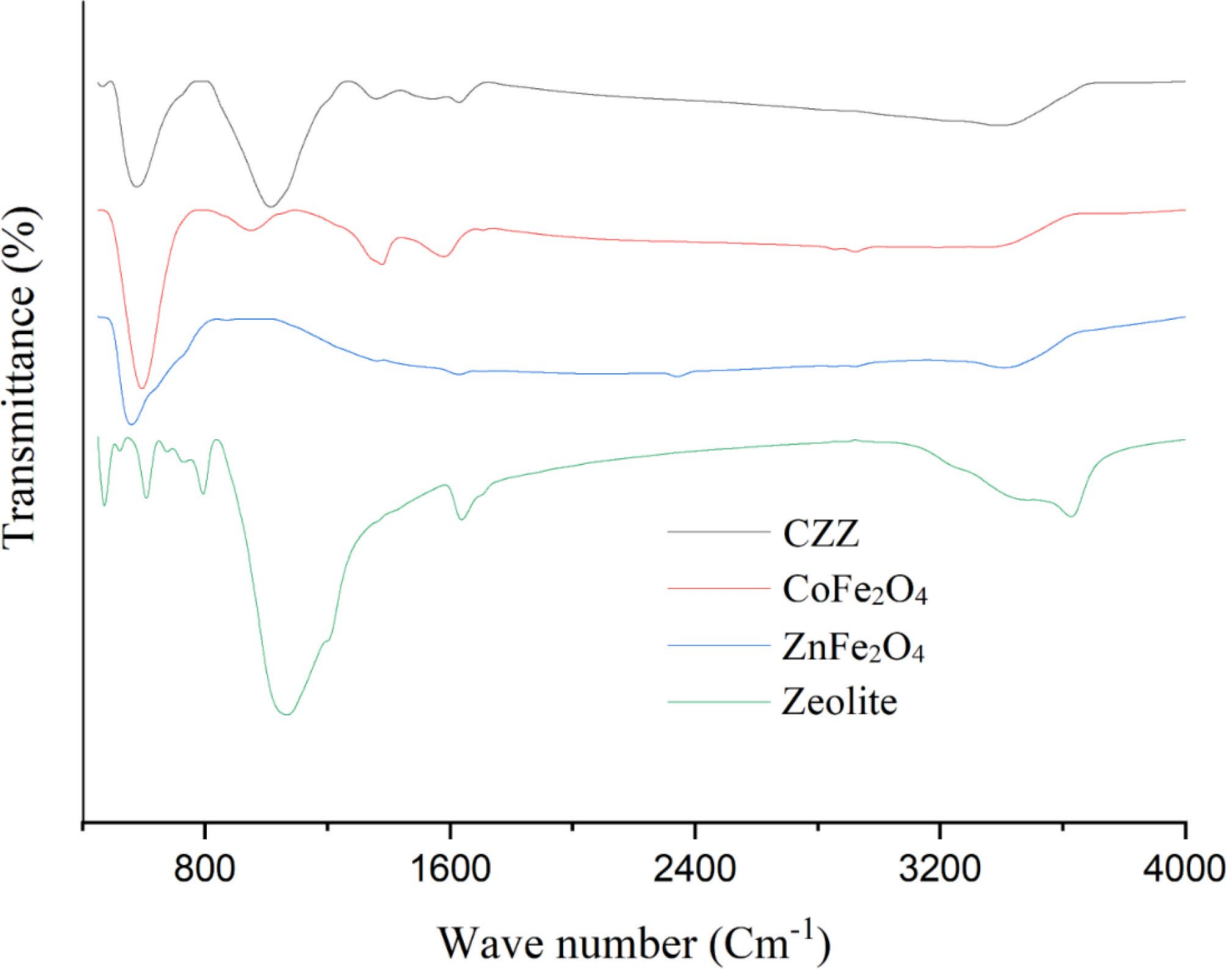
FTIR spectra of spinel ZnFe_2_O_4_ and CoFe_2_O_4_ nanoparticles, zeolite and CZZ.

FT-IR analysis was employed to determine the functional groups. The FT-IR spectra of the zeolite, ZnFe_2_O_4_, CoFe_2_O_4_ and CZZ are exhibited in Fig. 3. FT-IR spectra of all samples show the broad peak at around 3500 cm^− 1^ and a short band at around 1600 cm^− 1^ that are attributed to the hydroxyl stretching vibrations and the bending vibrations, respectively. In FT-IR spectra of zeolite and CZZ, the strong peak at 1050 cm^− 1^ is associated with stretching vibrations of the Si-O-Si bridge bonds in zeolite structure^45^. This peak in the FT-IR spectrum of CZZ is shorter than the zeolite spectrum due to the lower concentration of zeolite in the CZZ structure. In FT-IR spectra of ZnFe_2_O_4_, CoFe_2_O_4_ and CZZ, the strong band at about 580 cm^− 1^ is related to the stretching vibrations of Co–O, Fe-O and Zn–O bonds^46^. It is noteworthy that although the synthesis of CZZ occurs during chemical reactions, the absence of any additional peaks in the FTIR spectra indicates the complete formation of phases and proper washing of the samples.

**Fig. 4:**
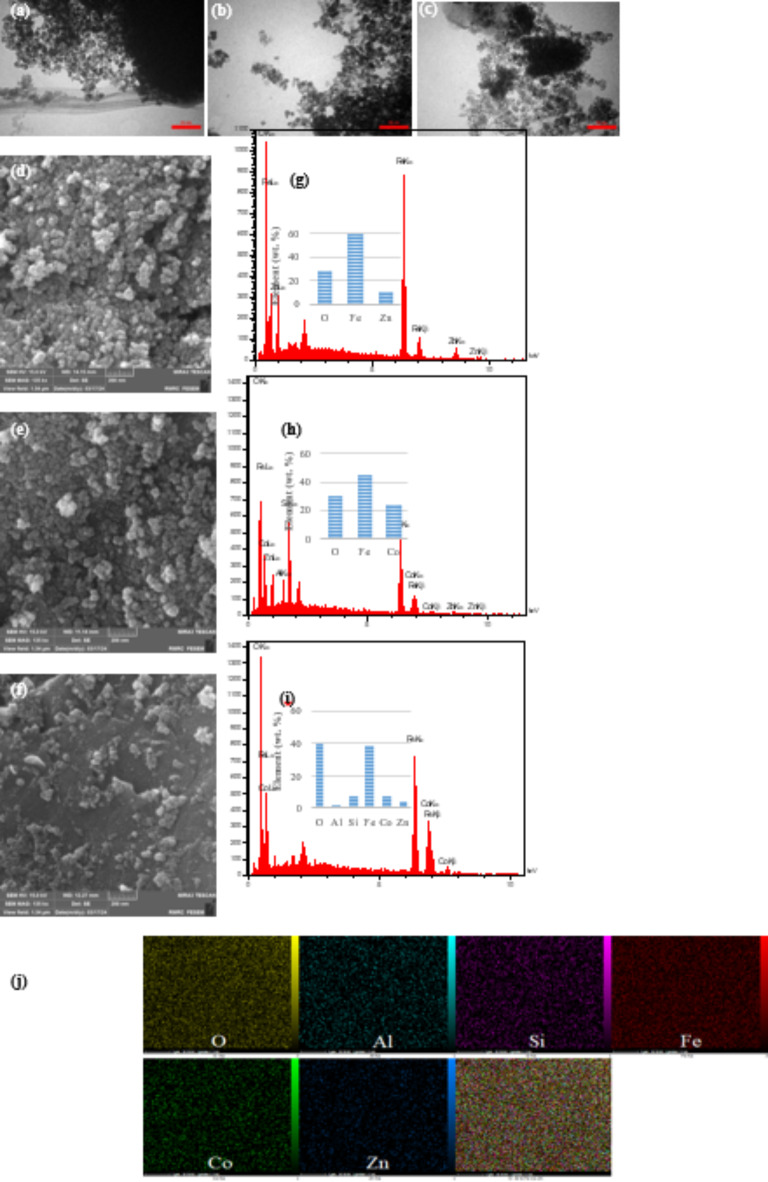
TEM images of (**a**) ZnFe_2_O_4_, (**b**) CoFe_2_O_4_ and (**c**) CZZ; FE-SEM images of (**d**) ZnFe_2_O_4_, (**e**) CoFe_2_O_4_ and (**f**) CZZ, EDS spectra of (**g**) ZnFe_2_O_4_, (**h**) CoFe_2_O_4_ and (**i**) CZZ and (**j**) mapping profiles of CZZ.

The morphological properties of samples were characterized using TEM and FESEM (Fig. 4). TEM images show that nanoparticles are spherical and slightly agglomerated (Fig. 4a, b). Agglomeration in nanoparticles that have magnetic properties is entirely predictable. Nevertheless, despite the strong magnetic properties and van der Waals forces between the particles due to the proper synthesis method, there is little agglomeration between the nanoparticles. After decorating the nanoparticles on the zeolite, the aggregation decreased somewhat (Fig. 4c). The reducing of accumulation leads to better and more complete access of pollutants to the surface of the nanocatalyst, and after that, there is a stunning increase in efficiency. Based on TEM images, the size of CoFe_2_O_4_ and ZnFe_2_O_4_ nanoparticles is about 5 and 8 nm, respectively. After immobilization of nanoparticles on the zeolite, a sharp decrease in the size of nanoparticles is observed, so the size of nanoparticles in CZZ is about 1.5–2 nm. The undeniable role of zeolite in morphology modulation is so apparent, that the size of nanoparticles in CZZ is remarkably reduced compared to single nanoparticles. Also, a narrow size distribution of nanoparticles is observed after anchoring. The smaller size of the nanoparticles due to the increase of the specific surface area causes more contact of pollutant molecules with the activated sites and subsequently increases the photocatalytic activity. The smaller size of nanoparticles and less agglomeration of nanoparticles after stabilization on zeolite can also be seen in FESEM images (Fig. 4d–f). EDS analysis reveals that the primary constituent elements of ZnFe_2_O_4_ and CoFe_2_O_4_ are zinc and cobalt, respectively, in addition to oxygen and iron (Fig. 4g,h). In the EDX spectrum of ZnFe_2_O_4_ and CoFe_2_O_4_, only the prominent peaks of the constituent elements are observed, which indicates the high purity of these compounds. In the spectrum of CZZ, in addition to Zn, Co, Fe, and O elements, characteristic peaks of zeolite can also be seen (Fig. 4i). Mapping analysis of CZZ in Fig. 4j displays that Co, Zn, and Fe elements are homogeneously distributed on zeolite support.

**Fig. 5:**
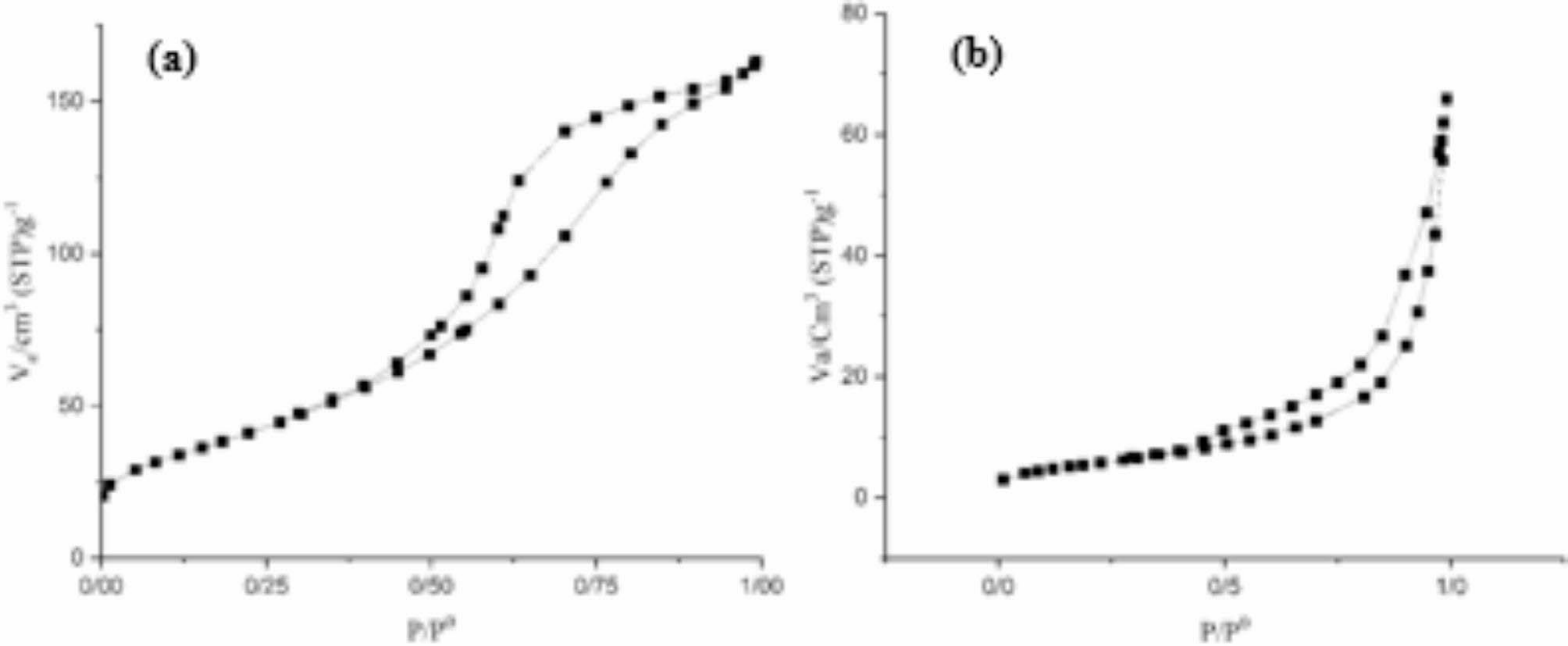
N_2_ adsorption/desorption isotherm of (**a**) CZZ and (**b**) zeolite.

To study the textural properties of samples, N_2_ adsorption-desorption was carried out. As revealed in Fig. 5, the N_2_ adsorption-desorption isotherms of all samples are demonstrated type IV isotherms with an H3 hysteresis loop, confirming the presence of a mesoporous structure in the photocatalysts^47^. High absorption of N_2_ adsorption–desorption isotherms of CZZ near P/P_0_ = 1 confirms the large size of mesopores. BET surface area, total pore volume, and average pore diameter of CZZ obtain 150.36 $\:{\text{m}}^{2}\:{\text{g}}^{-1}$, 0.2508$\:{\:\text{c}\text{m}}^{3}\:{\text{g}}^{-1},$ and 6.6717 nm, respectively. These values for zeolite are 20.394$\:{\:\text{m}}^{2}\:{\text{g}}^{-1}$, 0.1008 $\:{\:\text{c}\text{m}}^{3}\:{\text{g}}^{-1},$ and 19.771 nm, respectively. The specific surface area of CZZ is 7.5 times greater than that of bare zeolite. In general, nanoparticles are known for their high specific surface area due to their small size. Higher specific surface area is due to the immobilization of ZnFe_2_O_4_ and CoFe_2_O_4_ nanoparticles on zeolite sheets. The larger specific surface area means that the semiconductors on the CZZ surface are more likely to come in contact with pollutant molecules. Also, CZZ possesses a larger pore volume and a smaller average pore diameter than bare zeolite. Larger pore volume allows for better adsorption capacity and thus improves photocatalytic performance.

**Fig. 6:**
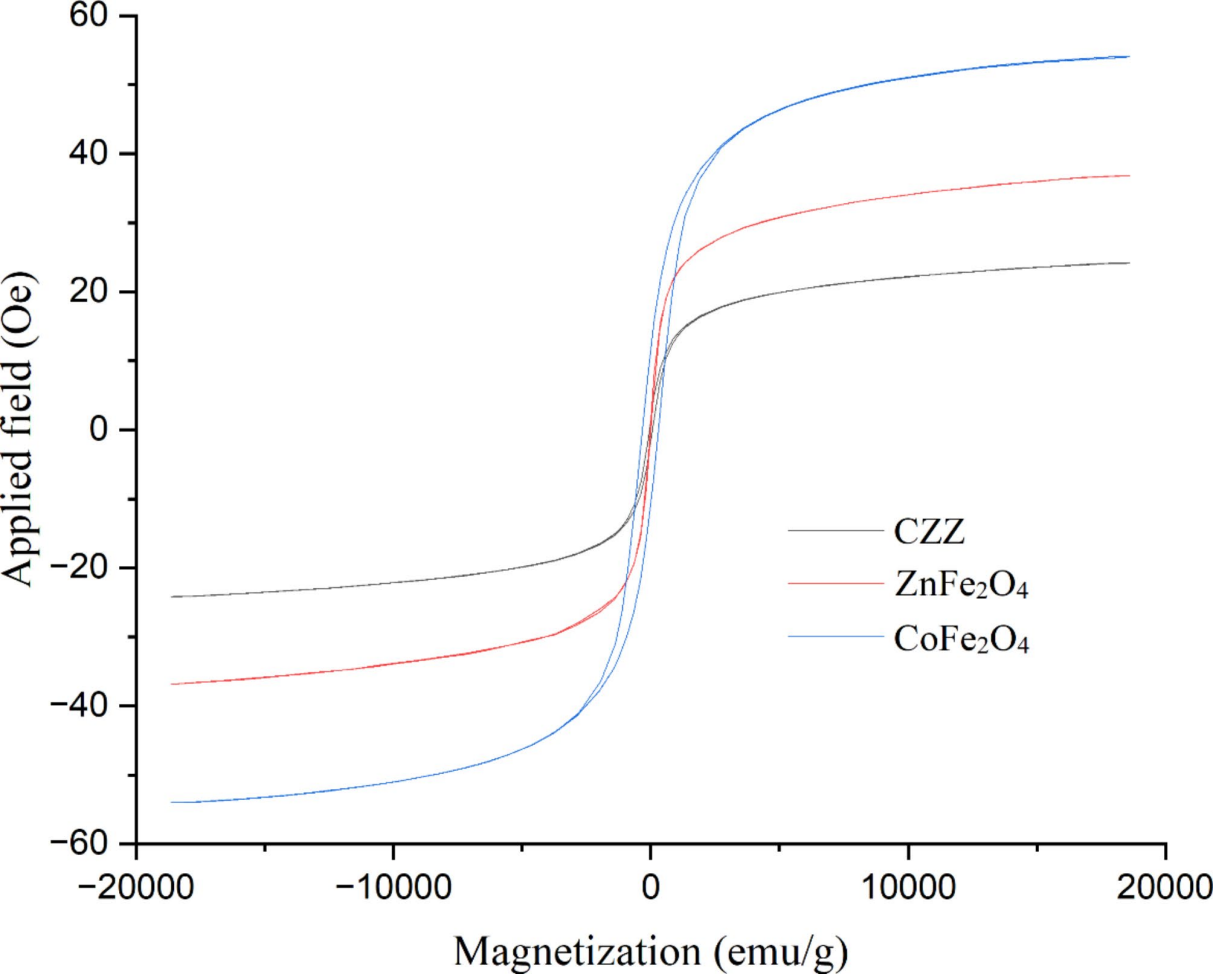
VSM of spinel ZnFe_2_O_4_ and CoFe_2_O_4_ nanoparticles and CZZ.

The magnetic hysteresis curve of samples was obtained using the VSM technique. As exhibited in Fig. 6, all samples show superparamagnetic behavior specified by the absence of hysteresis, remanence, and coercivity^48^. The saturation magnetization values (Ms) of bare ZnFe_2_O_4_ and CoFe_2_O_4_ are 36.8 and 54.0 emu/g, respectively. After immobilizing ZnFe_2_O_4_ and CoFe_2_O_4_ on zeolite support, the saturation magnetization of CZZ decreases to 24.4 54.0 emu/g. This reduction is due to a non-magnetic support in the CZZ structure. CZZ still displays a considerate behavior in the existence of a magnetic field. CZZ is simply separated from the reaction mixture using a magnet without producing secondary pollutants. This extraordinary feature allows CZZ to be utilized in industry as a recyclable catalyst.

**Fig. 7:**
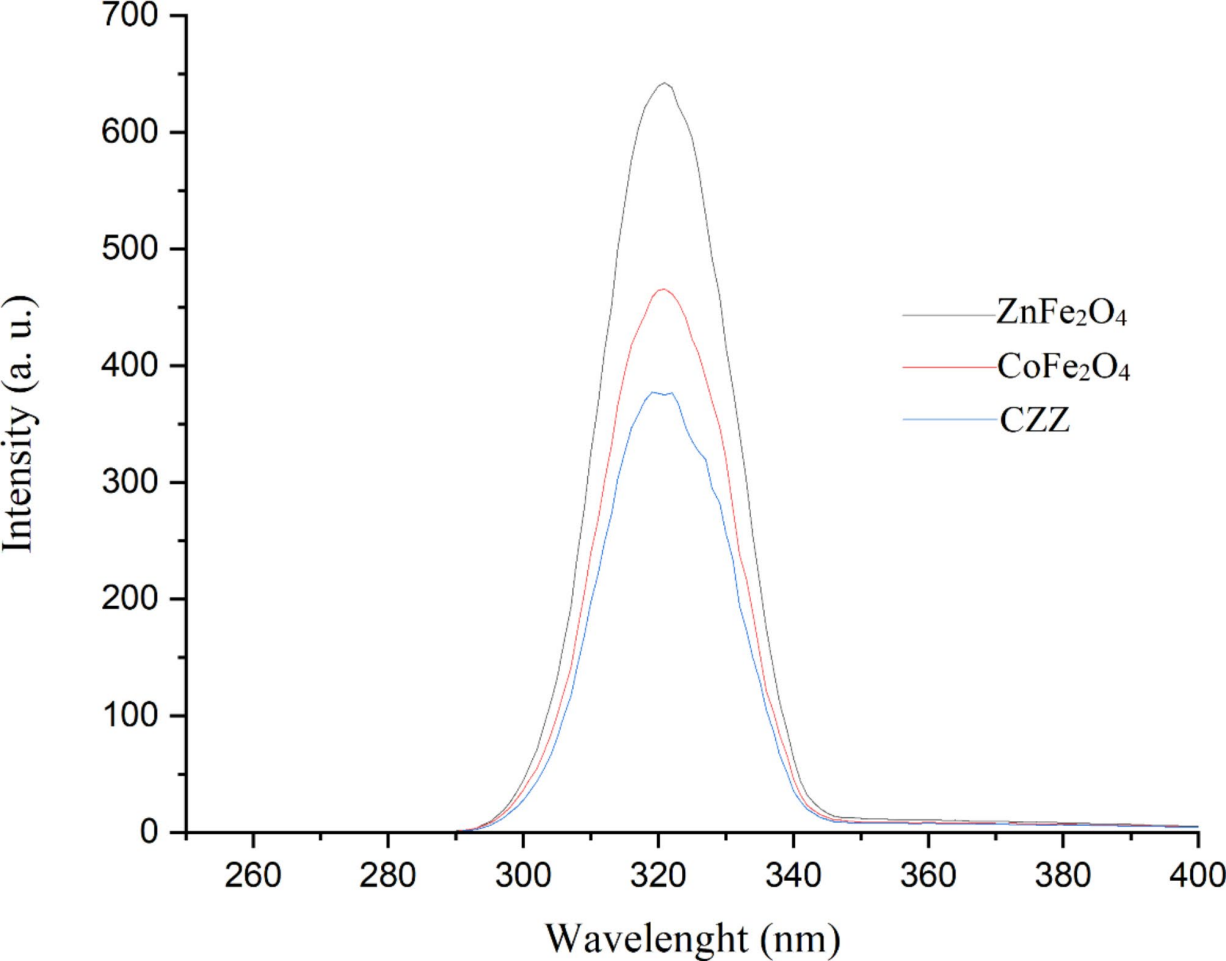
PL spectra of spinel ZnFe_2_O_4_ and CoFe_2_O_4_ nanoparticles and CZZ.

Photoluminescence (PL) analysis was used to evaluate the separation efficiency of photogenerated carriers in the photocatalyst. It is known that extended peak in the PL spectrum means higher recombination efficiency of photogenerative carriers in the photocatalyst and lower degradation efficiency. As demonstrated in Fig. 7, bare ZnFe_2_O_4_ and CoFe_2_O_4_ nanoparticles reveal strong PL emission peaks, while the intensity of PL emission peak of CZZ heterojunction was reduced. These observations propose that heterojunction in CZZ facilitates electron and hole transfer and helps to reduce undesirable charge recombination. In general, heterojunction fabrication helps improve carrier separation efficiency^49^. It is expected that the yield of Cr(VI) reduction in the presence of CZZ is higher than that of single nanoparticles.

**Fig. 8:**
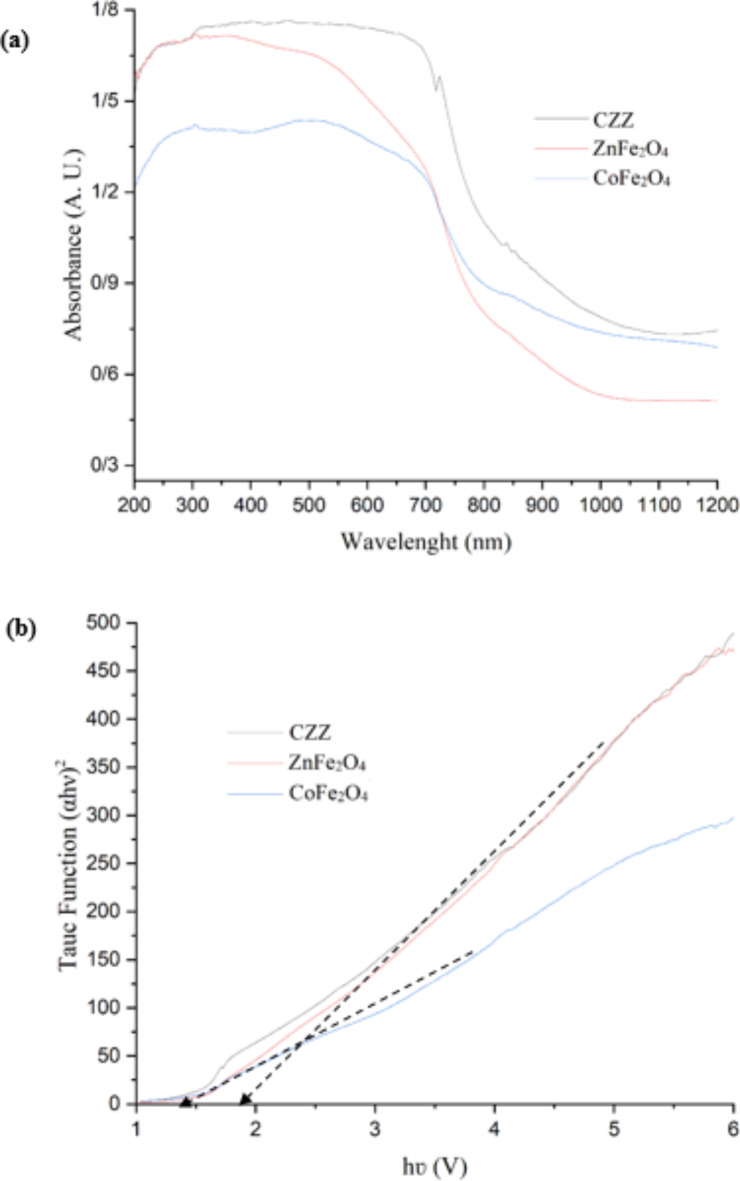
(**a**) UV-Vis DRS and (**b**) Tauc’s plots of spinel ZnFe_2_O_4_ and CoFe_2_O_4_ nanoparticles and CZZ.

The optical feature and band gap energy of the samples were evaluated using UV–vis DRS^50^ within the range of 200–1200 nm. According to Fig. 8a, all specimens show broad light absorption in both UV and Vis ranges. The intensity of the absorption peak in the case of CZZ is more potent than that of bare nanoparticles. The absorption Extension of UV–Vis in the heterojunction photocatalyst can lead to more favorable photocatalytic activity. Also, due to this broad absorption spectrum in the UV and visible range, renewable sunlight could activate the catalyst instead of expensive lamps with a short lifespan. On the other hand, according to Tauc’s plot (Fig. 8b), the bandgap energies of ZnFe_2_O_4_ and CoFe_2_O_4_ are 2.0 and 1.5 V, respectively. The Mulliken electronegativity formulas were used to calculate the energies of the conduction band ($\:{\text{E}}_{\text{C}\text{B}}$) and valance band ($\:{\text{E}}_{\text{V}\text{B}})$:

1 $$\:{\text{E}}_{\text{V}\text{B}}=\frac{{E}_{g}}{2}+\:{\upchi\:}-\:{\text{E}}_{\text{C}}$$

2 $$\:{\text{E}}_{\text{C}\text{B}}=\:{\text{E}}_{\text{V}\text{B}}-\:{\text{E}}_{\text{g}}$$

where, $\:{\text{E}}_{\text{C}}$ is the energy of free electrons (~ 4.5 V) on the hydrogen scale and χ is absolute electronegativity of semiconductors. χ is 5.05 eV for ZnFe_2_O_4_^51^ and 5.81 eV for CoFe_2_O_4_^52^. $\:{\text{E}}_{\text{C}\text{B}}\:\text{a}\text{n}\text{d}\:{\text{E}}_{\text{V}\text{B}}$ of ZnFe_2_O_4_ are − 0.45 and 1.55 V, respectively. These values ​​for CoFe_2_O_4_ are 0.56 and 2.06 V, respectively.

### Photocatalytic activity study

**Fig. 9:**
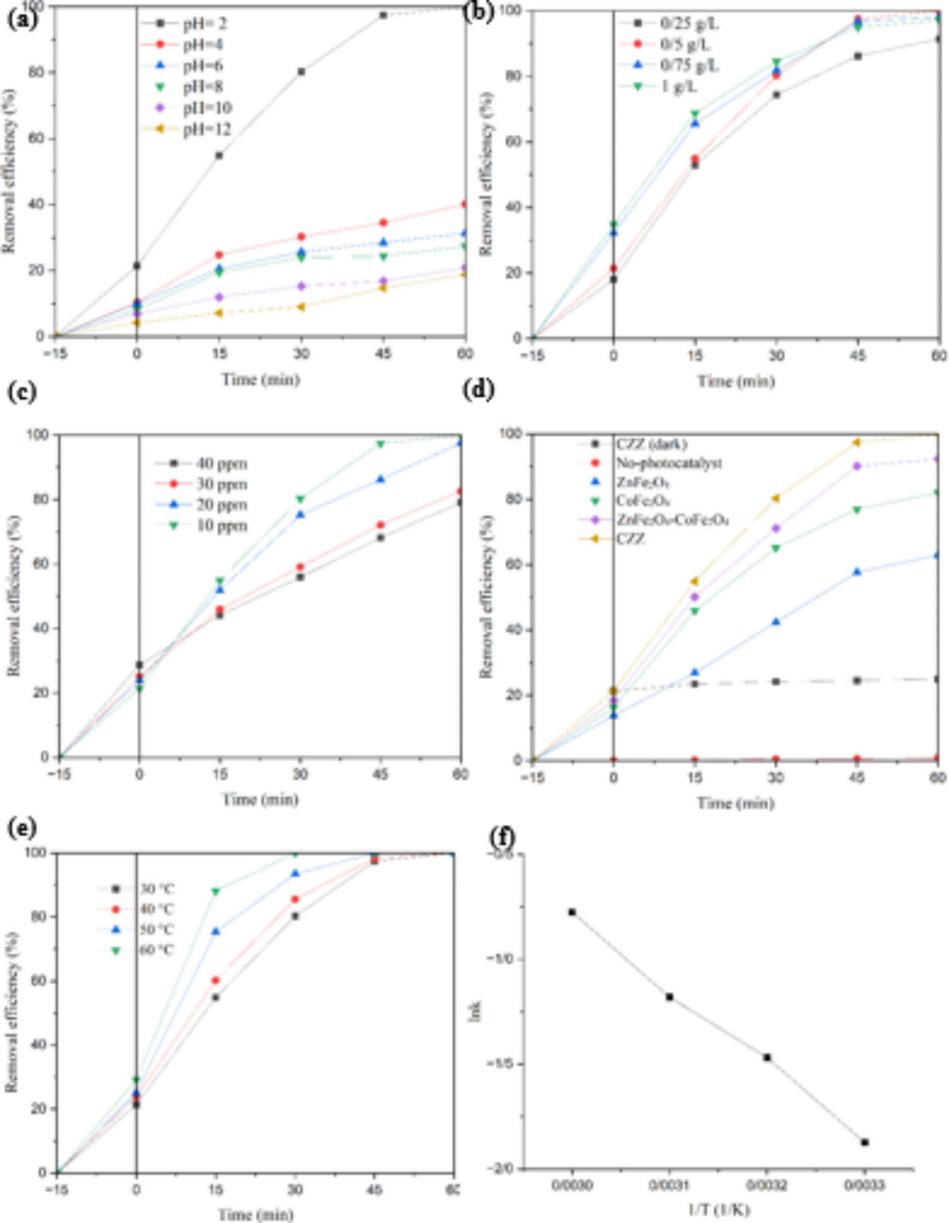
Effect of (**a**) pH initial; (**b**) photocatalyst dosage; (**c**) initial concentration of Cr(VI); (**d**) different catalysts on reduction Cr(VI); (**e**) temperature, and (**f**) relationship between reaction constant and temperature.

The photocatalysis behavior of CZZ heterojunction is studied by Cr(VI) photo-reduction under direct sunlight. At first, the effect of the initial pH value on the efficiency of catalytic reduction of Cr(VI) using CZZ heterojunction is studied. In general, photocatalytic reactions are strongly affected by the pH of the solution, and the acid-base state of the reaction system can have an essential influence on the progress of the reaction. As displayed in Fig. 9a, the photocatalytic reduction efficiency of Cr(VI) is significantly different under various pH values. The reduction efficiency increases considerably with decreasing pH. The Cr(VI) removal efficiency in the presence of CZZ increases from 18.8 to 100%, with pH adjustment from 12 to 2. The acidic environment is favorable for the Cr(VI) photoreduction due to the presence of H^+^ ions which promotes the chemical equilibrium to generate Cr(III). Cr(VI) is mainly present in $\:{\text{C}\text{r}}_{2}{\text{O}}_{7}^{-2}$ and $\:{\text{H}\text{C}\text{r}}_{2}{\text{O}}_{7}^{-}$under acidic conditions and according to the reactions (3 and 4), Cr(III) is produced as a result of the reduction reaction, while $\:{\text{C}\text{r}\text{O}}_{4}^{2-}$is mainly exists under alkaline conditions, and the product of its reduction reaction is $\:{\text{C}\text{r}\left(\text{O}\text{H}\right)}_{3}$ precipitation (5).

3 $${\text{C}}{{\text{r}}_2}{\text{O}}_7^{2 - } + 14{{\text{H}}^ + } + 6{{\text{e}}^ - } \to 2{\text{C}}{{\text{r}}^{3 + }} + 7{{\text{H}}_2}{\text{O}}$$

4 $${\text{HC}}{{\text{r}}_2}{\text{O}}_7^ - + 7{{\text{H}}^ + } + 3{{\text{e}}^ - } \to {\text{C}}{{\text{r}}^{3 + }} + 4{{\text{H}}_2}{\text{O}}$$

5 $${\text{CrO}}_4^{2 - } + 4{{\text{H}}_2}{\text{O}} + 3{{\text{e}}^ - } \to {\text{Cr}}{({\text{OH}})_3} + 5{\text{O}}{{\text{H}}^ - }$$

This result is confirmed based on the following findings:

First, based on Le Chatelier’s principle, the conversion of Cr(VI) to Cr(III) is better performed in the presence of more $\:{\text{H}}^{+}$ ions^4^. Second, the CZZ surface is positively charged in the acidic environment, further increasing the electrostatic attraction with Cr(VI) ions. Third, as the solution pH increases, the deposition of Cr(III) species on the CZZ surface increases, leaving fewer surface active sites available for adsorption, resulting in a decrease in efficiency.

To specify the optimum dosage of the photocatalyst for Cr(VI) reduction, a series of photocatalytic experiments with different dosages of CZZ in the range of 0.25–1 g/L are carried out at pH = 2. As revealed in Fig. 9b, as the catalyst dosage increases from 0.25 to 0.5 g/L, the Cr(VI) removal efficiency enhances from 91.43 to 100%, respectively. The possible reason for the increase in yield can be related to the rise in the number of active sites on the CZZ surface (ZnFe_2_O_4_ and CoFe_2_O_4_ nanoparticles), which leads to the improvement of the Cr(VI) reduction reaction. On the other hand, when the dose of CZZ is higher than 0.5 g/L, the reduction efficiency of Cr(VI) decreases. At photocatalyst higher doses, the limitation of light penetration and absorption should not be neglected due to the effect of light scattering in the reaction mixture. Thus, the appropriate dosage of CZZ is selected 0.5 g/L.

The Cr(VI) initial concentration is another critical factor in photocatalytic reduction. A series of tests on the Cr(VI) photocatalytic reduction at different initial concentrations of Cr(VI) is performed to investigate the effect of this parameter (Fig. 9c). The influence of Cr(VI) concentration on the photocatalytic activity of CZZ is examined at initial Cr(VI) concentrations from 10 to 40 ppm (at pH = 2 and dosage catalyst = 0.5 g/L). As expected, the photocatalytic performance of CZZ decreases from complete removal to 79.12% when the Cr(VI) concentration increases from 10 to 40 ppm. At higher concentrations of chromium (VI), the photons reaching the catalyst surface are limited^53^.

To study the effect of the catalyst in the photocatalytic reaction (Fig. 9d), the reduction reaction of Cr(VI) is investigated in the absence of CZZ at pH = 2. Without any catalyst, the reduction reaction of Cr(VI) is undetectable. Also, the effect of light and the amount of pollutant adsorption is studied. The reaction is investigated in the presence of CZZ in darkness for 60 min. The result of this experiment determines that about 25% of Cr(VI) is adsorbed on the surface of CZZ under dark conditions. The Cr(VI) removal curves in the presence of bare CoFe_2_O_4_ and ZnFe_2_O_4_ nanoparticles and CoFe_2_O_4_–ZnFe_2_O_4_ nanocomposite are shown in Fig. 9d. The reduction yields of Cr(VI) obtained 81.99%, 62.98% and 92.35% for bare CoFe_2_O_4_ and ZnFe_2_O_4_ nanoparticles and CoFe_2_O_4_–ZnFe_2_O_4_ nanocomposite, respectively. A pseudo-first order model is performed to fit the kinetic plots of photocatalytic Cr(VI) reduction^54^. CZZ shows the highest k (0.1535 min^− 1^), which is 5.37, 9.27, and 3.58 times higher than CoFe_2_O_4_ (0.0286 min^–1^), ZnFe_2_O_4_ (0.0166 min^–1^) and CoFe_2_O_4_-ZnFe_2_O_4_ (0.0428 min^–1^), respectively. Also, turn over frequency (TOF) of samples is calculated using the following formula:

$$\:TOF=\:\frac{n}{m\:\times\:t}$$

where n is the moles number of reduced Cr(VI), m is the mass of photocatalyst and t is reaction time. TOF for reduction of Cr(VI) using CoFe_2_O_4_, ZnFe_2_O_4_, CoFe_2_O_4_–ZnFe_2_O_4_ and CZZ is found 5.2$\:\times\:{10}^{-6}$, 4.0$\:\times\:{10}^{-6}$, 5.9$\:\times\:{10}^{-6}$ and 6.4$\:\times\:{10}^{-6}$ mol/g min, respectively. This result was consistent with the obtained results from TEM and SEM analysis. Smaller size of nanoparticles and more availability of nanoparticles lead to increase Cr(VI) reduction efficiency. Combining zeolite with semiconductors can be one of the reasons for improving the efficiency due to the occurrence of positive synergistic effects that help to solve the lack of structure. On the other hand, immobilization of nanoparticles on zeolite prevents their agglomeration, increases the specific surface area of ​​nanoparticles, and ultimately leads to an increase in degradation efficiency. In addition to these, the formation of an internal electric field between CoFe_2_O_4_ and ZnFe_2_O_4_ can effectively promote carrier separation and increase the photocatalytic oxidation-reduction efficiency.

The effect of temperature on the reduction of Cr(VI) is also examined. As shown in Fig. 9e, the temperature has a considerable impact on the photoreduction efficiency of Cr(VI), so that with the increase in temperature, the time of complete reduction of Cr(VI) decreases. In the following, the value of k is calculated at different temperatures. The value of k is 0.4605, 0.3070, 0.2303 and 0.1535 min^− 1^ at 60, 50, 40 and 30 °C, respectively. At a higher temperature, the movement of molecules and the number of their collisions increases, and as a result, the reaction efficiency is enhanced. Natably, the relationship between k and temperature is investigated using the Arrhenius formula:

$$\:lnk=lnA-\:\frac{{E}_{a}}{RT}$$

where, k, A, E_a_, R and T are the reduction reaction constant, the pre-exponential (frequency) factor, the activation energy, the molar gas constant (8.314 J/mol.K) and temperature, respectively. The E_a_ is calculated as 29.79 kJ/mol according to the results revealed in Fig. 9f.

The practicality of CZZ photocatalyst in reducing Cr(VI) in different aqueous media is tested. The tap water, river water and seawater are performed instead of distilled water to prepare Cr (VI) aqueous solutions. The photocatalytic processes are expected to proceed with disruption due to the presence of foreign co-existing ions in different aqueous environments. Interestingly, the interfering ions have not effect on the reduction reaction of Cr(VI) in this catalytic system. This result indicates excellent anti-interference ability of CZZ.

**Fig. 10:**
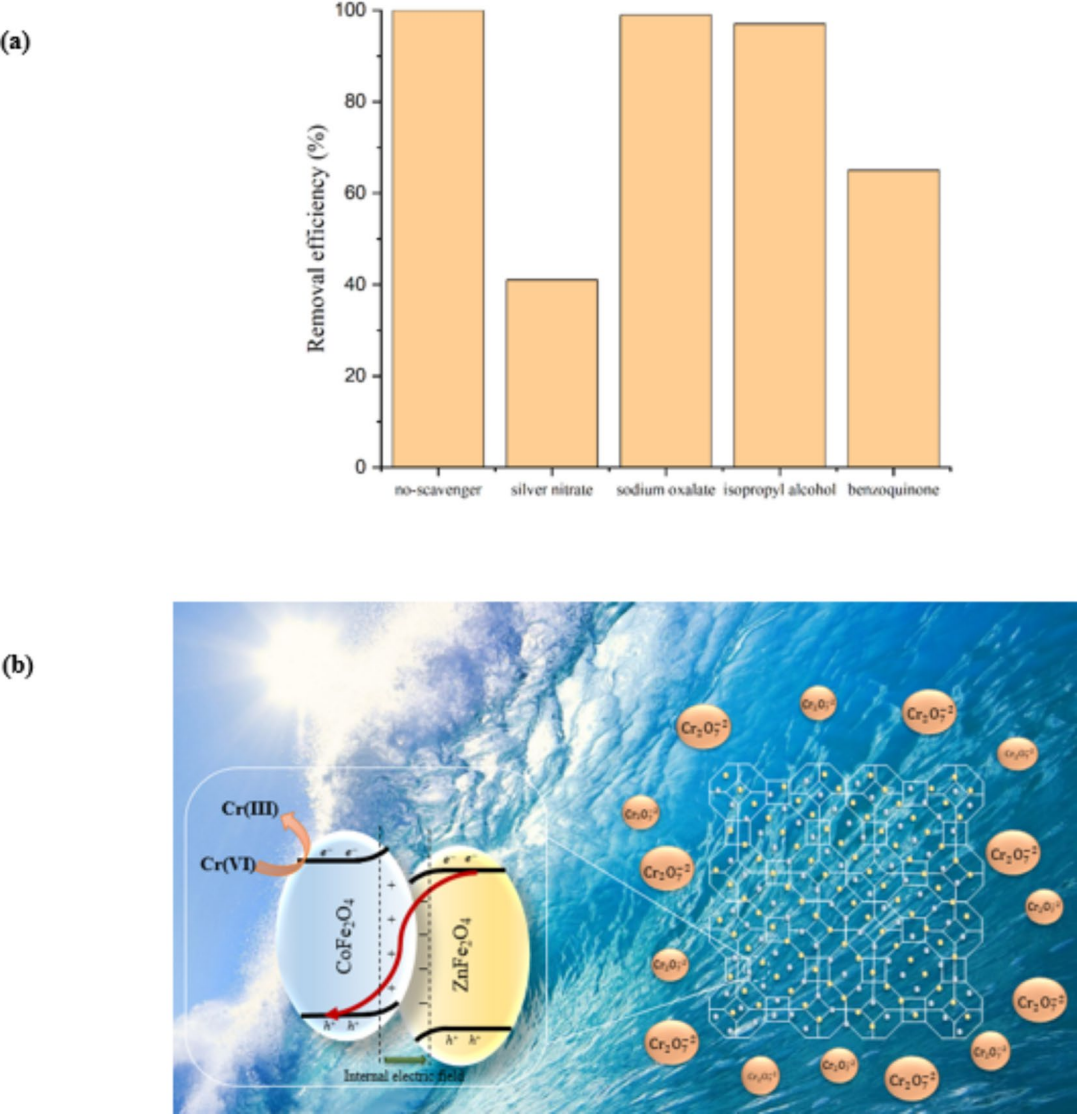
(**a**) Scavenger tests and (**b**) proposed photoreduction mechanism of Cr(VI) reduction over CZZ under sunlight.

Also, AgNO_3_ and formic acid are performed as electron scavenging agents, to study the mechanism of Cr(VI) reduction using CZZ. Yield of Cr(VI) removal decreases sharply in the presence of electron scavenging agent. In addition to examining scavenging agents, the effect of other scavengers on the efficiency of Cr(VI) reduction in the catalytic system is studied and exhibited in Fig. 10a. Sodium oxalate^55^, isopropyl alcohol^56^, and benzoquinone^57^ are famous scavenging agents for trapping h^+^, ^Ÿ^OH, and $\:{\text{O}}_{2}^{\bullet\:-}$ radicals, respectively. As displayed in Fig. 10a, h^+^ and ^Ÿ^OH do not contribute to the photocatalytic reduction of Cr(VI). According the vital role of electron and $\:{\text{O}}_{2}^{\bullet\:-}$ in this photocatalytic system, a mechanism for reduction of Cr(VI) is proposed below.

Band energy structures are essential to understand light-induced interfacial transport of charge carriers and to discern the mechanism. When two semiconductors are in contact, it is necessary to investigate the charge transfer mode in the photocatalytic process. After light irradiation and stimulation of the photocatalyst, electrons are excited. If reaction mechanism follows the conventional heterojunction, photo-generated electrons from the CB of ZnFe_2_O_4_ migrate to the CB of CoFe_2_O_4_ due to the CB position of ZnFe_2_O_4_ (− 0.45 V) is more negative than that of the CoFe_2_O_4_ (+ 0.56 eV). This phenomenon led to the absence of $\:{\text{O}}_{2}^{\bullet\:-}$ radical production, because CB of CoFe_2_O_4_ was more positive than E^0^(O_2_/$\:{\text{O}}_{2}^{\bullet\:-}$). On the other hand, excited electrons have a high tendency to immediately recombine with holes, which reduces the efficiency of the photocatalyst. In heterojunction structures, the energy bands of semiconductors are bent up and down based on $\:{\text{E}}_{\text{C}\text{B}}$ and $\:{\text{E}}_{\text{V}\text{B}}$ values. According to DRS analysis of samples, electrons in lower $\:{\text{E}}_{\text{C}\text{B}}$ of ZnFe_2_O_4_ can combine with holes in lower $\:{\text{E}}_{\text{V}\text{B}}$ of CoFe_2_O_4_ and improve the separation efficiency of electron-hole pairs (Fig. 10b). Some of the photogenerated electrons in CB ZnFe_2_O_4_ can react with oxygen molecules to produce $\:{\text{O}}_{2}^{\bullet\:-}$, because the potential of CB ZnFe_2_O_4_ (-0.45 V) is lower than E^0^ O_2_/$\:{\text{O}}_{2}^{\bullet\:-}$ = − 0.33 V vs. NHE^16^. The generated radicals are responsible for the reduction of Cr(VI), Still, most of the Cr(VI) is directly reduced by the electrons in the CB of ZnFe_2_O_4_ beacuse its potential is negative than the E^0^ Cr(VI)/Cr(III) = 1.33 V vs. NHE^58^. The prominent role of electrons and $\:{\text{O}}_{2}^{\bullet\:-}$ radicals in Cr(VI) reduction is also determined by performing scavenger tests. Therefore, light-induced electrons accumulate on the CZZ heterojunction surface, which is beneficial for the converting Cr(VI) to Cr(III). On the other hand, considering that the VB of CoFe_2_O_4_ is more negative than the E^0^ (H_2_O/^•^OH), the produced holes in VB are unable to react with water molecules to produce hydroxyl radicals. This result was understandable even from the scavenging tests and the minimal contribution of h^+^ to the reduction reaction. In some reports, EDTA is used as a hole scavenger agent^31^. At the beginning of our work, we investigated the reduction reaction of Cr(VI) in the presence of EDTA and the results were almost the same as when EDTA was not present. Fortunately, in this catalytic system, the holes did not disturb the photocatalytic reaction, and there was no need to trap the holes. The reaction of electron-hole pair production, radicals generation, and reduction of Cr(VI) is described by the following equations:

$$\:\text{C}\text{Z}\text{Z}+\text{h}\text{v}\:\to\:\:{\text{e}}^{-}+\:{\text{h}}^{+}$$

$$\:{\text{O}}_{2}+\:{\text{e}}^{-}\to\:\:{\text{O}}_{2}^{{\bullet\:}-}$$

$$\:\text{C}\text{r}\left(\text{V}\text{I}\right)+\:{\text{e}}^{-}\:\to\:\text{C}\text{r}\left(\text{I}\text{I}\text{I}\right)$$

$$\:\text{C}\text{r}\left(\text{V}\text{I}\right)+\:{\text{O}}_{2}^{{\bullet\:}-}\:\to\:\text{C}\text{r}\left(\text{I}\text{I}\text{I}\right)$$

**Fig. 11:**
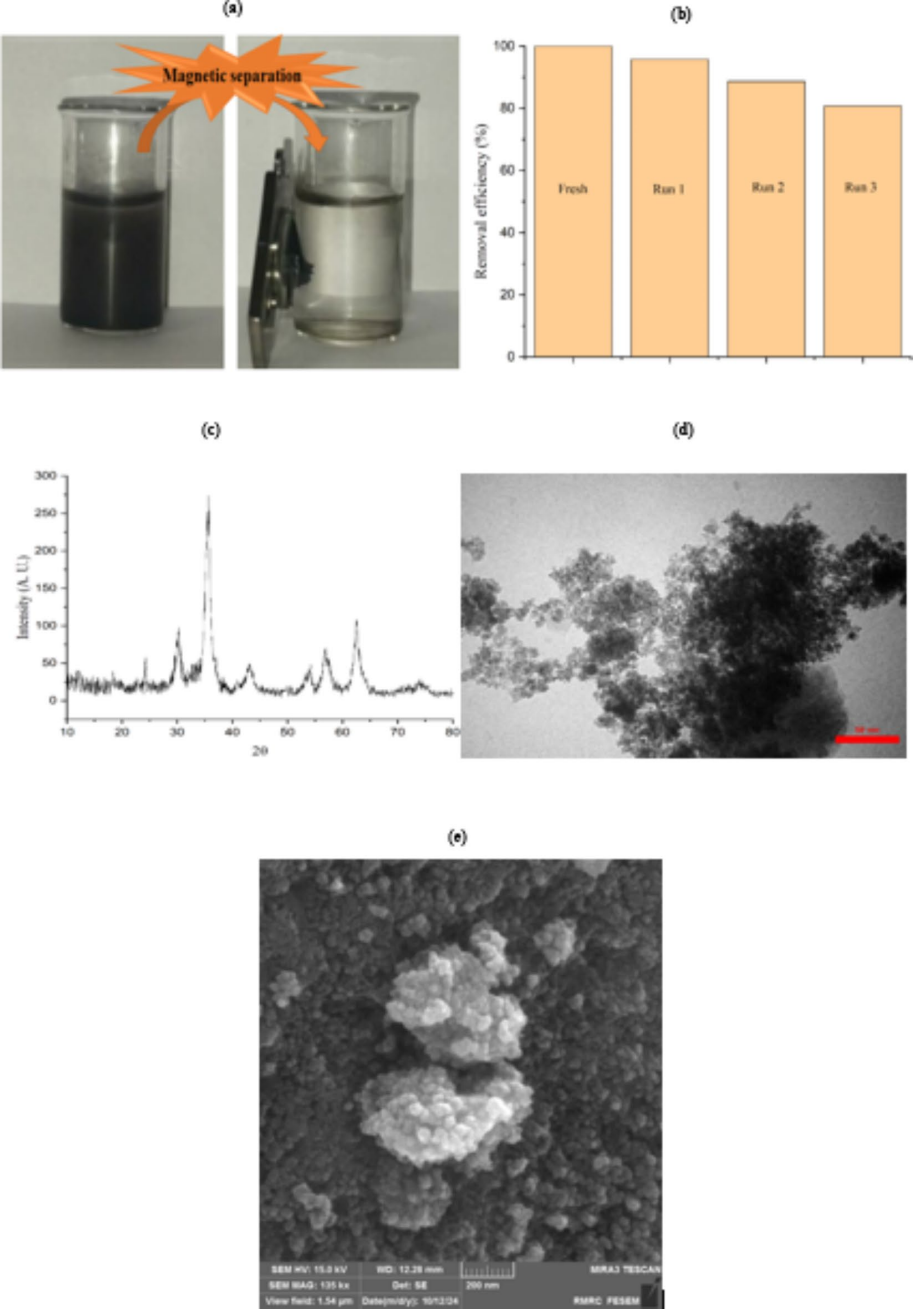
(**a**) Magnetic separation of CZZ; (**b**) The recycle tests for CZZ towards Cr(VI) reduction; (**c**) XRD spectrum; (**d**) TEM image and (**e**) FE-SEM image of reused CZZ.

Having suitable structural and chemical stability is an essential factor for a photocatalyst. So, the chemical stability of the CZZ photocatalyst is examined through three consecutive reduction reactions of Cr(VI). The CZZ is separated from the reaction medium using an external magnet (Fig. 11a), washed several times with water and ethanol, and dried, and a sufficient amount is utilized as a photocatalyst for the next run. After three runs, the reduction efficiency of Cr(VI) remains above 80%, indicating excellent regeneration performance (Fig. 11b). Also, TOF value for the reduction of Cr(VI) using the recovered CZZ is obtained 1.11$\:\times\:{10}^{-6}$ mol/g min. Compared to the fresh catalyst, this amount is acceptable. XRD is employed to check the influences of recycling on the crystalline phases of CZZ and reveals that successive reuse does not cause any detectable changes (Fig. 11c). Also, from the comparison of the TEM image (Fig. 4c) and FESEM (Fig. 4f) of the fresh catalyst and the recycled catalyst (Fig. 11d, e), it can be concluded that the reaction environment does not affect the morphology and size of the nanoparticles. CZZ has sufficient stability and can withstand the reaction conditions without noticeable change. This valuable result shows that CZZ has significant stability under the tested experimental conditions.

To highlight the advantages of reduction of Cr(VI) in this catalytic system, the results of this study were compared with some previous reports and are displayed in Table 1. As shown in Table 1, CZZ reduces Cr(VI) in a shorter time without using any auxiliary agent. In some cases, the TOF of the current work is lower than the previous work. Still, easy magnetic separation instead of time-consuming filtrations and using sunlight renewable energy instead of expensive lamps are the main advantages of this catalytic system.

**Table 1:** Comparison of the efficiency of Cr(VI) reduction in various systems.

Catalyst (dosage, g)	Time (min)	Catalyst separation	Auxiliary agent	Source of stimulation	V(mL), C (mg/L) of Cr(VI)	TOF (mol/g min)	T (°C)	Efficiency (%)
Bi_2_MoO_6_/Bi_2_S_3_ (0.03)	120	Filtering	–	Xe lamp	100, 20	9.4$\:\times\:{10}^{-6}$	25	88^53^
illite-g-C_3_N_4_ (0.03)	60	Filtering	EDTA-2Na	Xe lamp	40, 20	7.8$\:\times\:{10}^{-6}$	at^*^	91.8^58^
CF/C_3_N_4_/Bi_2_MoO_6_(nm^**^)	90	Filtering	–	Xe lamp	100, 50	–	22 ± 2	80^59^
Ag@Ag_3_VO_4_/AgPMo(nm)	150	Centrifugation	EDTA-2Na	Xe lamp	50, 4	–	at	94.8^32^
SnS_2_/La_2_Ti_2_O_7_(0.3)	270	nm	–	Xe lamp	300, 50	3$\:0.4\times\:{10}^{-6}$	at	95^60^
UNiMOF/BiVO_4_/S-C_3_N_4_(0.03)	120	Centrifugation	–	Xe lamp	70, 30	1.7$\:\times\:{10}^{-5}$	at	93.6^22^
Ti_3_C_2_/Bi_2.15_WO_6_ (0.05)	120	Centrifugation	–	Xe lamp	50, 10	1.5$\:\times\:{10}^{-6}$	at	92.5^61^
Bi_2_S_3_-BiVO_4_-G(0.05)	120	Filtering	–	Xe lamp	50, 50	8.0$\:\times\:{10}^{-6}$	25 ± 2	~ 100^62^
NiFe_2_O_4_/Bi/Bi_2_WO_6_/Bi_5_O_7_I (0.05)	60	Centrifugation	–	LED lamp	100, 25	1.5$\:\times\:{10}^{-5}$	at	95^63^
C-doped BiOCl/Bi_2_ S_3_ (0.05)	120	nm	–	Xe lamp	30, 50	4.8$\:\times\:{10}^{-6}$	at	99.5^5^
TiO_2_ film	180	Filtering	Fe (III)	UV-LED	nm	–	25 ± 1	95.8^64^
TiO_2_ (0.05)	90	Filtering	–	UV lamp	100, 20	8.5$\:\times\:{10}^{-6}$	at	99^19^
BiVO_4_/FeVO_4_@rGO(0.03)	100	nm	–	Xe lamp	50, 20	5.7$\:\times\:{10}^{-6}$	at	90.9^55^
Ag/C–TiO_2_/Cd_0.5_Zn_0.5_S(0.05)	120	Filtering	–	Xe lamp	100, 5	1.5$\:\times\:{10}^{-6}$	at	95.5^65^
NH_2_-UiO-66 (Zr) (0.01)	120	Centrifugation and filtering	–	Xe lamp	50, 20	1.6$\:\times\:{10}^{-5}$	at	99.5^66^
BiPO_4_/CuBi_2_O_4_(0.02)	90	nm	–	Xe lamp	50, 30	9.6$\:\times\:{10}^{-6}$	at	60.3^67^
Cu-doped ZnAl_2_O_4_(0.1)	150	Centrifugation and filtering	–	Sunlight	100, 1000	9.6$\:\times\:{10}^{-5}$	30	75^68^
CoAl-LDH/TiO_2_(nm)	150	nm	–	Xe lamp	10, 10	–	at	94.7^69^
CZZ(0.025)	60	Magnetic separation	–	Sunlight	50, 10	$\:6.4\:\times\:{10}^{-6}$	at	This study

*Ambient temperature.

**Not Mentioned.

## Conclusion

Herein, the development of a solar photocatalytic process using a ternary magnetic heterojunction nanocatalyst for the effective reduction of Cr(VI) was successfully carried out. The combination of ZnFe_2_O_4_ and CoFe_2_O_4_ nanoparticles with zeolite was an effective strategy to increase photocatalytic performance. The effects of five operating conditions such as adsorption, type of catalyst, dosage of CZZ, initial Cr(VI) concentration, and initial pH value were tested experimentally. The outstanding efficiency of Cr(VI) reduction was obtained at about 100% after 60 min reaction with E_a_ = 29.79 kJ/mol under optimized conditions (pH = 2, dosage catalyst = 0.5 g/L, and initial concentration of Cr(VI) = 10 ppm). The practical application of CZZ photocatalyst in different aqueous environments was examined. A high potential was observed for reducing Cr(VI) in tap water, seawater, wastewater and river water due to the excellent anti-interference ability of CZZ. The recycling experiments confirmed excellent stability of CZZ photocatalyst. Quench experiments revealed the vital role of electron and $\:{\text{O}}_{2}^{\bullet\:-}$ in the photoreduction of Cr(VI) over CZZ, and based on the obtained results, the S-scheme mechanism was proposed. Along with many advantages for CZZ photocatalyst, such as high efficiency in photocatalytic reaction, good mechanical and chemical durability, non-toxicity, easy magnetic separation from reaction mixture can be introduced as a strong point for this catalytic system. This study significantly contributes to our understanding of the design of ternary photocatalysts using S-scheme transfer for the removal of hazardous pollutants and also to obtain new ideas for the degradation of toxins from real wastewater.

## Data Availability

The authors declare that the data supporting the findings of this study are available within the paper files. Should any raw data files be needed in another format they are available from the corresponding author upon reasonable request.
